# Predicting Hotspots for Influenza Virus Reassortment

**DOI:** 10.3201/eid1904.120903

**Published:** 2013-04

**Authors:** Trevon L. Fuller, Marius Gilbert, Vincent Martin, Julien Cappelle, Parviez Hosseini, Kevin Y. Njabo, Soad Abdel Aziz, Xiangming Xiao, Peter Daszak, Thomas B. Smith

**Affiliations:** University of California, Los Angeles, California, USA (T.L. Fuller, K.Y. Njabo, T.B. Smith);; Université Libre de Bruxelles, Brussels, Belgium (M. Gilbert);; Food and Agriculture Organization of the United Nations, Beijing, People’s Republic of China (V. Martin);; Centre de Cooperation International en Recherche Agronomique pour le Developpement, Montpellier, France (J. Cappelle);; EcoHealth Alliance, New York, New York, USA (P. Hosseini, P. Daszak);; National Laboratory for Quality Control on Poultry Production, Dokki, Giza, Egypt (S.A. Aziz);; University of Oklahoma, Oklahoma City, Oklahoma, USA (X. Xiao)

**Keywords:** influenza in birds, influenza A virus H3N2 subtype, influenza A virus H5N1 subtype, reassortant viruses, viruses, zoonoses, avian influenza, influenza

## Abstract

The 1957 and 1968 influenza pandemics, each of which killed ≈1 million persons, arose through reassortment events. Influenza virus in humans and domestic animals could reassort and cause another pandemic. To identify geographic areas where agricultural production systems are conducive to reassortment, we fitted multivariate regression models to surveillance data on influenza A virus subtype H5N1 among poultry in China and Egypt and subtype H3N2 among humans. We then applied the models across Asia and Egypt to predict where subtype H3N2 from humans and subtype H5N1 from birds overlap; this overlap serves as a proxy for co-infection and in vivo reassortment. For Asia, we refined the prioritization by identifying areas that also have high swine density. Potential geographic foci of reassortment include the northern plains of India, coastal and central provinces of China, the western Korean Peninsula and southwestern Japan in Asia, and the Nile Delta in Egypt.

## Abstract 2

TOC summary: Reassortment is most likely to occur in eastern China, central China, or the Nile Delta in Egypt.

Simultaneous infection with multiple influenza virus strains can affect virus fitness components, such as virus growth performance, and thus affect virus pathogenicity, transmission, or recombination (*1*). In a host infected with 2 closely related influenza viruses, the stains can reassort, exchanging gene segments to produce new strains, some of which might have increased virulence. Virulence might also trade off with transmission such that more pathogenic viruses spread more slowly (*2*). However, in some instances, a reassortant virus can have high transmissibility and high pathogenicity. For example, reassortment between influenza viruses of humans and birds resulted in the 1957 and 1968 pandemic viruses, each of which is estimated to have killed ≈1 million persons (*3*,*4*). The exchange of genes between pairs of influenza virus subtypes increased virulence in animal models, including reassortment between subtypes H9N2 and H1N1, between H5N1 and H1N1, and between H3N2 and H5N1 (*5*,*6*). We focus on reassortment between subtypes H3N2 and H5N1 because extensive data are available, but given sufficient data, our approach could be extended to other subtypes.

For seasonal influenza virus A subtype H3N2, person-to-person transmissibility and prevalence among humans are high (*7*). Furthermore, subtype H5N1, which is primarily found in birds, can be highly pathogenic; the fatality rate among humans is 60% (*8*). In mice, ≈8% of reassortant viruses formed from human subtype H3N2 and avian subtype H5N1 resulted in increased virulence and a mortality rate of 100% (*5*). This finding among mice raises the possibility that among humans reassortment events between subtypes H3N2 and H5N1 could generate a novel influenza virus that could spread rapidly, resulting in many deaths. To prioritize areas where future reassortment is most likely to occur, we analyzed surveillance data for subtype H5N1 among poultry in the People’s Republic of China and Egypt and subtype H3N2 among humans. We chose China and Egypt because both countries have had recent outbreaks of subtype H5N1 infection among poultry, human deaths from subtype H5N1 infection, and extensive spatial data on cases of infection with subtype H5N1. This information would help decision makers implement policies to reduce spillover in these areas (*9*). Areas with high risk for co-occurrence of these 2 influenza virus subtypes along with high densities of susceptible hosts, such as swine, quail, or turkeys, could benefit from enhanced monitoring and farm and market biosecurity.

## Materials and Methods

### Influenza Data

#### Egypt

All data for Egypt were aggregated to the scale of the markaz, an administrative district that includes several villages. The dataset for subtype H5N1 infections during 2009–2012 consisted of 453 cases among poultry in backyard flocks, farms, and live-bird markets in 35 markazes. Screening assays are described elsewhere (*10*). See Technical Appendix Figure 1, for a workflow analysis. Most (72%) positive samples came from chickens in backyard flocks. The available geographic data on subtype H3N2 in Egypt are limited (Technical Appendix Table 3), so we used human population density as a surrogate for cases of infection with subtype H3N2 because virtually all humans (except those who have been vaccinated or infected recently) will be susceptible to infection with subtype H3N2.

**Figure 1:**
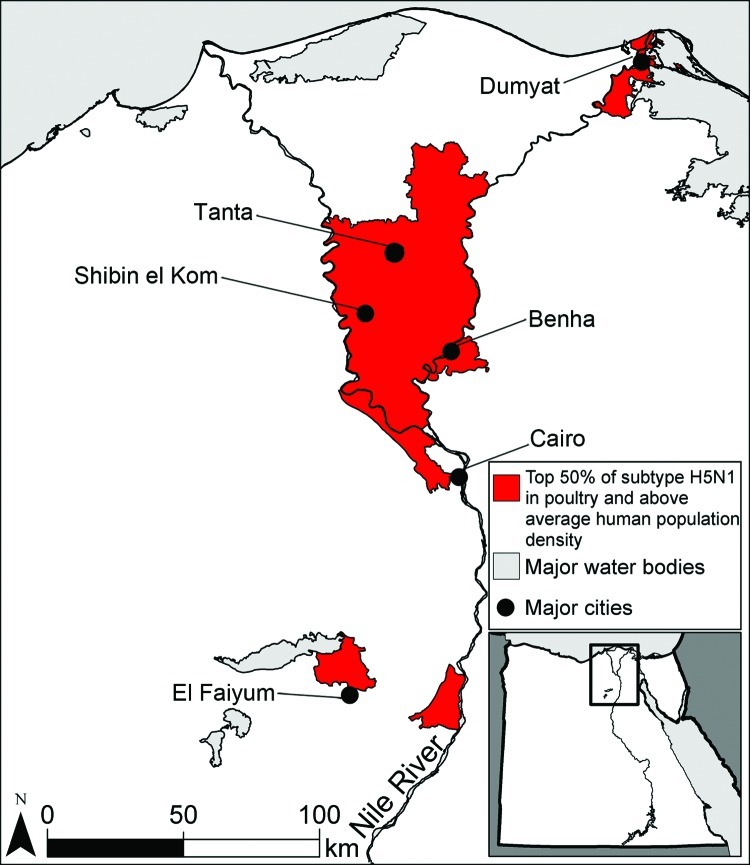
Potential influenza reassortment areas in Egypt. Districts in red are predicted to have an above average number of cases of influenza subtype H5N1 virus in poultry and an above average human population density, which is a proxy for subtype H3N2 virus infections.

#### China

All data for China were aggregated to the spatial scale of the prefecture, an administrative unit within a province that typically contains several towns and villages. We examined 2 independent datasets for cases of subtype H5N1 infection on the basis of outbreaks on poultry farms and active surveillance of live-bird markets (*11*). The data on cases of subtype H3N2 infection were retrieved by querying GenBank and the EpiFlu database available from the Global Initiative on Sharing All Influenza Data (GISAID) website (http://platform.gisaid.org) for all occurrences of subtype H3N2 in China that included fine-scale geographic data on the prefecture in which the sample was collected (Technical Appendix Table 1). Data on subtype H3N2 cases were available for 35 prefectures and comprised 632 human cases collected over 14 years. However these data are limited because in a typical influenza year in China, hundreds of millions of cases might occur. Therefore, we also compared the GenBank and GISAID data on subtype H3N2 cases with human population density, assuming that the density is a proxy for the true number of subtype H3N2 cases.

### Ecologic Variables

#### Subtype H3N2

For China, we predicted the probability of occurrence of subtype H3N2 cases by using environmental factors hypothesized in previous studies to be major drivers of human influenza: human population density, percentage urban area, precipitation, and temperature (Technical Appendix Table 2). For instance, we incorporated population into the model because we hypothesized that human influenza cases would be more likely to occur in high-density urban areas with a large number of susceptible human hosts (*12*). For Egypt, we used human population density as a proxy for subtype H3N2 infections.

#### Subtype H5N1

To predict occurrence of subtype H5N1 cases, we used the following as environmental covariates: chicken and duck density, human population density, percentage of each prefecture occupied by bodies of water, and percentage of cultivated cropland per prefecture or markaz (Technical Appendix Table 2). These variables were included because results of previous studies have associated them with risk for subtype H5N1 (*11*,*13*,*14*). For example, human population was included as a predictor of subtype H5N1 because it serves as an indirect measure of intensity of poultry trade (*15*). For Egypt, we used overall poultry density because density of chickens and ducks separately was not available.

### Swine Density

To identify potential areas of influenza reassortment, we refined the co-occurrence maps by also incorporating swine density. We used density data from the Food and Agriculture Organization of the United Nations, which constructed these data by extrapolating from agricultural censuses and livestock surveys by using regression models (*16*). The rationale was to focus on areas where subtypes H3N2 and H5N1 might exchange genes in livestock because swine support co-infections with multiple lineages of the influenza virus, which occasionally generate novel strains (*17*,*18*).

### Statistical Models

#### Egypt

We constructed a Poisson regression model in which the dependent variable represents the count of a rare event. The dependent variable was the number of cases of subtype H5N1 in poultry per district. The independent variables were poultry density, human population density, percentage cropland, and percentage water per district. The dataset included sites where poultry were negative for subtype H5N1. We identified districts predicted to have an above average number of cases of subtype H5N1 among poultry and above average human population density. Such districts could be the site of double infections with subtypes H3N2 and H5N1 in humans and of in vivo reassortment.

#### China

We used multivariate logistic regression to relate occurrence of subtypes H3N2 and H5N1 to the aforementioned ecologic variables (Technical Appendix Table 2). The logistic regression models were built by using population density, percentage urban area, temperature, and precipitation as predictors of subtype H3N2 presence and chicken and duck density, human population density, percentage cropland per prefecture, and percentage water as predictors of subtype H5N1 presence. The datasets comprise occurrences of subtypes H5N1 or H3N2 but lack negative occurrences. Therefore, we selected negative sites at random and then fitted a logistic regression model to the positive and random negative occurrences. To reduce the bias of random negative occurrences, we selected negative sites at random 10,000× and calculated the average of the parameters of the logistic regression model over these randomizations.

## Results

### Egypt

Areas with a high number of cases of subtype H5N1 in poultry and high human population density, which we used as a surrogate for subtype H3N2 infections in humans, were located in the Nile Valley and Delta in Lower Egypt (Figure 1). These areas could be sites of human co-infection with subtypes H3N2 and H5N1, leading to the evolution of novel influenza strains. Major cities located within 10 km of potential reassortment hotpots are Benha, Cairo, Dumyat, El Faiyum, and Shibin el Kom, which could be prioritized for increased surveillance to detect reassortment events and prevent spread. Poultry density per district was a highly statistically significant predictor of subtype H5N1 in poultry (Table), probably because high bird densities facilitate transmission of the virus among flocks in a village. The percentage of cropland per district was highly correlated with poultry density (ρ = 0.72), so we included only the latter in the regression model. The percentage of water per district also approached significance, which could be because family compounds in rural areas where backyard flocks are raised are typically located near canals and irrigated fields.

**Table:** Effect of environmental variables on occurrence of influenza virus subtypes*

Location, subtype (data source)	Coefficient	SE	p value
China			
Subtype H3N2			
Intercept	−5.184	0.946	**8.7 × 10^−8^**
Human population	9.47 × 10^−4^	3.23 × 10^−4^	**4.47 × 10^−2^**
Percentage urban	0.113	0.117	0.284
Precipitation	4.59 × 10^−3^	8.87 × 10^−3^	0.573
Temperature	1.24 × 10^−2^	8.27 × 10^−3^	0.195
Subtype H5N1 (surveillance dataset)			
Intercept	−7.93	1.66	**3.01 × 10^−5^**
Chicken density	−6.94 × 10^−2^	0.438	0.572
Duck density	1.53	0.433	**3.45 × 10^−3^**
Human population	1.41 × 10^−3^	2.85 × 10^−4^	**1.77 × 10^−4^**
Percentage agriculture	2.18 × 10^−4^	1.52 × 10^−4^	0.218
Percentage water	7.32 × 10^−2^	1.7 × 10^−2^	**2.36 × 10^−3^**
Subtype H5N1 (outbreak dataset)			
Intercept	−9.24	1.14	**3.15 × 10^−12^**
Chicken density	1.24	0.277	**2.87 × 10^−4^**
Duck density	0.542	0.24	0.117
Human population	1.37 × 10^−3^	2.67 × 10^−4^	**1.41 × 10^−3^**
Percentage agriculture	2.13 × 10^−4^	8.84 × 10^−5^	5.4 × 10^−2^
Percentage water	0.101	1.91 × 10^−2^	**4.53 × 10^−3^**
Egypt			
Subtype H5N1			
Intercept	1.83	0.516	**4 × 10^−4^**
Poultry density	7.86 × 10^−4^	2.31 × 10^−4^	**7 × 10^−4^**
Human population	3.36 × 10^−2^	6.49 × 10^−2^	0.605
Percentage water	0.752	0.41	6.64 × 10^−2^
*Boldface indicates p<0.05.			

### China

Cases of subtype H3N2 in humans in China were mostly concentrated along the east coast (Technical Appendix Figure 2, panel A). The association between subtype H3N2 and human population density was significant (Table). Human population density, climate, and the percentage of urban areas per prefecture collectively explained ≈60% of the risk for subtype H3N2 occurrence (*R*^2^ = 0.596, area under the curve [AUC] = 0.902). Subtype H3N2 is expected to occur primarily in central, eastern, and southern China (Technical Appendix Figure 2, panel B). In the surveillance and the outbreak datasets, statistically significant drivers of subtype H5N1 occurrences were human population, duck density, and percentage of water. The models for subtype H5N1 had moderate predictive power (*R*^2^ surveillance = 0.604, AUC surveillance = 0.918, *R*^2^ outbreak = 0.424, AUC outbreak = 0.848).

**Figure 2:**
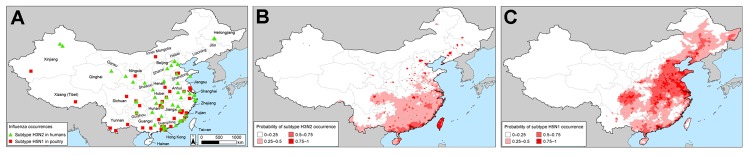
Influenza empirical data and occurrence maps for influenza virus subtypes H3N2 and H5N1. A) Observed cases of subtypes H3N2 and H5N1 in People’s Republic of China, according to outbreaks reported to the Chinese Ministry of Agriculture. B) Spatial model of the probability of subtype H3N2 at the prefecture scale predicted by using logistic regression. C) Risk for subtype H5N1 according to the outbreak dataset. See Technical Appendix Figure 2, for the corresponding map for the surveillance dataset.

**Figure 3:**
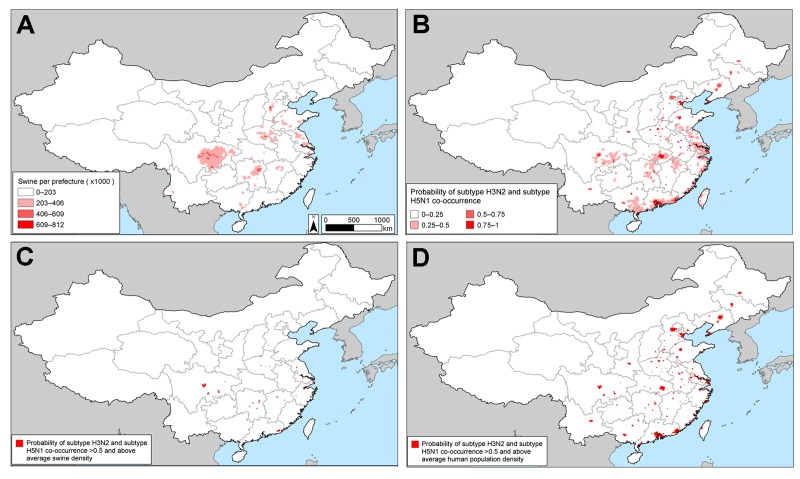
Potential influenza reassortment areas in People’s Republic of China determined by using the influenza virus subtype H5N1 outbreak dataset. A) Density of swine. B) Spatial model of the risk for subtype H3N2 and H5N1 co-occurrence according to the outbreak dataset. C) Areas with a probability of subtype H5N1 and H3N2 co-occurrence >50% and above average swine density. D) Areas with a probability of subtype H5N1 and H3N2 co-occurrence >50% and above average human population density. See Technical Appendix Figure 3, for corresponding maps based on the subtype H5N1 surveillance dataset.

After creating maps of the probability of occurrence of subtypes H3N2 and H5N1, we multiplied the maps by one another to predict the probability of co-occurrence of the 2 subtypes (Figure 3, panel B; Technical Appendix Figure 3, panel B). We classified an area as a potential reassortment hotspot if the probability of both subtypes occurring at the site was >50% and the density of swine was above average. Analysis of other swine density thresholds yielded similar results. The consensus of the spatial models (Figure 3, panel C; Technical Appendix Figure 3, panel C) is that in China, there are 2 main geographic foci of risk for reassortment of subtypes H3N2 and H5N1: 1) the coastal provinces bordering the South China Sea and East China Sea (Guangdong, Jiangsu, Shanghai, and Zhejiang Provinces) and 2) central China (Hunan and Sichuan Provinces). The added value of modeling areas where subtypes H3N2 and H5N1 co-occur versus modeling based exclusively on subtype H5N1 is that the former approach pinpoints a smaller geographic region that can be prioritized for increased surveillance or farm biosafety. Mapping areas based on the probability of subtype H5N1 occurrence alone would prioritize additional provinces to the southwest (Henan, Hebei, and Hubei) and to the north (Beijing, Hebei, Liaoning, and Tianjin) of the 6 provinces that we identified as potential areas for reassortment between subtypes H3N2 and H5N1 (Figure 2, panel C; online Technical Appendix Figure 2, panel C). Our prioritization of a smaller geographic area is valuable if the resources for surveillance are insufficient to enable sampling of all of the provinces that are at risk for subtype H5N1.

### East Asia

**Figure 4:**
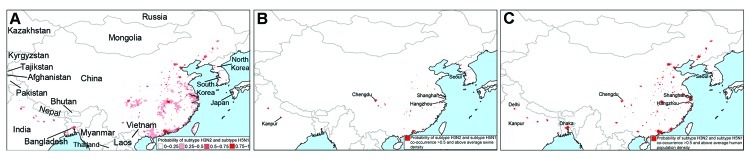
Reassortment areas elsewhere in Asia based on the People’s Republic of China model constructed from the influenza virus subtype H5N1 outbreak dataset. A) Probability of subtype H3N2 and H5N1 co-occurrence (according to the subtype H5N1 outbreak dataset). B) Areas with a probability of subtype H5N1 and H3N2 co-occurrence >50% and above average swine density. C) Areas with a probability of subtype H5N1 and H3N2 co-occurrence >50% and above average human population density. See Technical Appendix Figure 4, for corresponding models based on the surveillance dataset.

We applied the influenza virus subtypes H3N2 and H5N1 logistic regression models that were fitted to the data from China to neighboring countries for which chicken and duck density data were available (*19*). As in the analysis for China, we multiplied the subtype H3N2 and H5N1 models to predict areas of co-occurrence between the subtypes and overlaid swine density. To the extent that these areas have above average swine density and a >50% chance for co-occurrence of subtypes H3N2 and H5N1, potential reassortment hotspots are the northern plains of India (Uttar Pradesh), the western Korean Peninsula (Daejeon, Gyeonggi, Jeollabuk Provinces of South Korea and Pyonganbuk and Pyongannam Provinces of North Korea), and southwestern Japan (Saga Prefecture on Kyushu Island) (Figure 4, panel B; Technical Appendix Figure 4, panel B). Major cities with >500,000 persons near these hotspots include Kanpur, India; Chengdu, Sichuan, central China; Hangzhou and Shanghai, eastern China; and Seoul, South Korea. Risk is higher in these cities because they have high densities of swine, which could be a mixing vessel for reassortment of subtypes H5N1 and H3N2, and a high potential for infection with subtype H5N1 and H3N2 according to our regression models; for example, the models indicate that the ecologic suitability of Shanghai is 0.97 for subtype H3N2 and 0.996 for subtype H5N1. Incorporating population density as a proxy for infection with subtype H3N2 results in predictions that are compatible with the models based on swine density but also identifies 2 other megacities of >10 million persons that could be at high risk for virus reassortment: Dhaka, Bangladesh and Delhi, India (Figure 4, panel C; online Technical Appendix Figure 4, panel C).

## Discussion

The spatial models presented here predict that a reassortant influenza (H3N2/H5N1) virus is most likely to originate in the coastal and central provinces of China or the Nile Delta region of Egypt. The probability that subtypes H3N2 and H5N1 will co-occur in these regions is high (Figure 1; Figure 3, panel C; Technical Appendix Figure 4, panel C), which could lead to dual infection in mammalian hosts, such as swine or humans in China or humans in Egypt. Co-infection could subsequently result in in vivo reassortment. Although the influenza A(H1N1)pdm09 virus is hypothesized to have originated from Mexico (*20*), southern China remains a major hotspot for the generation of novel influenza viruses (*21*). Our spatial models are compatible with this longstanding observation insofar as we predict that the southern coastal province of Guangdong is a potential hotspot for the evolution of novel influenza viruses by reassortment.

A caveat is that even if virus subtypes H3N2 and H5N1 were to reassort in swine, the spread of the reassortant virus among humans might require further virus adaptation events; for example, mutations might be required for the virus to replicate efficiently in humans or to be transmitted among humans (*22*). Recent work has shown that as few as 5 aa substitutions are required for aerosol spread of subtype H5N1 among mammals (*23*). With these qualifications in mind, this analysis provides actionable recommendations about which areas to target for intensified farm and market surveillance. Such surveillance could enable early detection of a reassortant influenza (H3N2/H5N1) virus, should it arise in swine, and facilitate containment of the virus before it crosses the species barrier to humans.

Our finding that in China the probability of subtype H3N2 infection increases with human population density is compatible with previous studies that detected a positive association between population, influenza cases, and mortality rates (*12*,*24*). Reasons for this association could be that the number of susceptible human hosts increases with population (*11*) or that surveillance efforts are greater in populous areas (*25*). Our results with regard to subtype H5N1 in birds are also largely consistent with those of previous studies that mapped subtype H5N1 hotspots in China and Egypt. In China, several provinces identified as having high ecologic suitability for subtype H5N1 (including Shandong, Jiangsu, and Sichuan) were also identified as subtype H5N1 hotspots in a previous study that used a different statistical model and different predictor variables (*11*). In China, previous analyses have concluded that risk for subtype H5N1 increases with the density of domestic ducks (*26*). In Egypt, earlier studies identified high-intensity crop production as a statistically significant predictor of subtype H5N1 in poultry (*27*). Similarly, we found that subtype H5N1 infections in poultry were associated with poultry density, which was highly correlated with crop production. In a previous study, models constructed from satellite images of vegetation predicted that the highest environmental suitability for subtype H5N1 is along the Nile River and in the Nile Delta (*28*). Our models were constructed from different predictor variables, such as poultry density, but yielded similar results: the highest number of subtype H5N1 cases in poultry were predicted to occur in districts in the Nile Delta.

Efforts to contain the A(H1N1)pdm09 virus would have been more effective if the virus had been detected in animal populations before it was transmitted to humans (*29*). Continuous zoonotic influenza surveillance is needed in China and Egypt and requires a network of laboratories to screen surveillance samples and requires financial incentives to encourage poultry producers and sellers to report outbreaks. One strategy for early detection of a reassortant virus could involve increasing farm and market surveillance in the identified areas (i.e., live-bird markets in 6 provinces in China [Guangdong, Hunan, Jiangsu, Shanghai, Sichuan, and Zhejiang] that have a >50% chance of subtype H3N2 and H5N1 co-occurrence and above average swine density). Increased monitoring could identify hotspots where subtype H5N1 is circulating, leading to more efficient targeted vaccination of poultry, and could pinpoint prefectures at high risk for a reassortant virus. In China, sanitary practices, such as cage disinfection and manure disposal, would substantially reduce risk for subtype H5N1 in live-bird markets (*30*).

In Egypt, our results support increased surveillance of backyard flocks near Benha, Cairo, Dumyat, El Faiyum, Shibin el Kom, and Tanta, where suitability for subtypes H5N1 and H3N2 is predicted to be high. Control measures could include compensation plans and vaccination of poultry with a recently developed subtype H5N1 vaccine that is more effective than previous vaccines against strains circulating in Egypt (*10*). Reporting of poultry disease outbreaks in Lower Egypt is poor (*31*), probably because farmers fear loss of income if authorities cull their flocks. Indeed, birds suspected to be infected with subtype H5N1 are often sold quickly at a discount, resulting in virus transmission to buyers’ flocks and families (*32*). If equitable compensation schemes were implemented, reporting of subtype H5N1 might increase and outbreaks could be contained more quickly, reducing opportunities for subtypes H5N1 and H3N2 to co-infect humans or domestic animals and, thus, for reassortment.

In general, policies such as culling must have a scientific basis because these measures have major effects on the economy and animal welfare. For example, when part of a swine herd is culled to contain an outbreak, it might become necessary to euthanize the entire herd, including animals with no influenza exposure, because buyers will not accept them (*33*). Furthermore, influenza outbreaks among livestock can trigger major global declines in meat prices, and the nature and timing of veterinary health authorities’ responses to an outbreak can affect the extent to which demand recovers after the crisis. In particular, when control measures such as culling are scientifically well justified and explained to the public soon after the start of an outbreak, consumer confidence is restored more quickly (*34*).

Although our maps suggest a risk for reassortment in Lower Egypt and eastern and central China, in vivo reassortment of subtypes H3N2 and H5N1 has not been detected in humans in these areas. On the other hand, numerous infections with influenza (H3N2)v, a reassortant virus that contains genes from a subtype H3N2 virus circulating in swine and from the A(H1N1)pdm09 virus, have been detected in humans in North America (*35*,*36*). This finding raises the question of why subtype H3N2v has spread but subtype H3N2/H5N1 reassortants have not. Spread of subtype H3N2v could result from the fact that the reassortant virus contains the M gene from the A(H1N1)pdm09 virus, which increases aerosol transmission (*35*,*37*). Our models might explain why, in contrast with subtype H3N2v reassortants, no subtype H3N2/H5N1 reassortants have been detected in humans. For example, we predict that subtypes H3N2 and H5N1 occur in Hunan, China, a province that has high swine density and was the geographic origin of subtype H5N1 viruses in clade 2.1 (*38*). Influenza (H3N2/H5N1) reassortants in which the nonstructural gene comes from a clade 2.1 virus replicate poorly in mice (*5*). Thus, subtype H3N2/H5N1 reassortants might not have emerged as often as subtype H3N2v reassortants because the provinces where subtypes H3N2 and H5N1 overlap contain a clade of subtype H5N1, whose genes reduce the fitness of reassortant viruses. If this hypothesis is correct, if subtypes H5N1 and H3N2 infect a pig in central China and exchange genes, the hybrid virus might not replicate efficiently or transmit to other hosts. Furthermore, a reassortant virus with surface proteins similar to those of subtype H3N2 viruses that have circulated in humans recently might have poor transmissibility because of preexisting immunity (*18*).

Applying our modeling framework to other zoonotic influenza subtypes, such as H3N2v, could yield insight about geographic hotspots of reassortment and the pattern of spatial spread of reassortants. To accomplish this, 2 data limitations must be overcome. First, to be incorporated into spatial models, influenza sequences submitted to GenBank or GISAID should be accompanied by geographic data at relatively high spatial resolution, for example, names of cities or counties where sampling was conducted. However, such sequences are often accompanied by only the state or country of the sample, which reduces the usefulness of the data for fine-scale spatial modeling (*39*). For example, we searched online databases and confirmed that the geographic data available for Indonesia are insufficient to construct a spatial model to predict sites with a high risk for reassortment. Second, more extensive surveillance of livestock is needed to provide sufficient sample sizes to parameterize geographic models. Currently, the number of influenza subtype H1, H3, and H5 viruses from swine in major databases is an order of magnitude lower than that available for humans (Technical Appendix Table 3). Additional surveillance of swine could lead to better predictions about hotspots of influenza in livestock and sites of potential swine-to-human transmission. Livestock surveillance campaigns should sample large geographic areas and include regions where production is high (*35*). 

The potential for reassortment between human and avian influenza viruses underscores the value of a One Health approach that recognizes that emerging diseases arise at the convergence of the human and animal domains (*29*,*40*). Although our analysis focused on the influenza virus, our modeling framework can be generalized to characterize other potential emerging infectious diseases at the human–animal interface.

Technical Appendix Workflow of the analysis; influenza risk maps and empirical data; and prioritization of high-risk areas for influenza reassortment in People’s Republic of China.
