# Supplementary material for: Predicting Hotspots for Influenza Virus Reassortment
Source: Emerg Infect Dis. 2013 Apr;19(4):581–8. doi: 10.3201/eid1904.120903 (PMC3647410; doi:10.3201/eid1904.120903)
Supplement: Technical Appendix — Workflow of the analysis; influenza risk maps and empirical data; and prioritization of high-risk areas for influenza reassortment in People’s Republic of China. [file 12-0903-Techapp-s1.pdf]

Article DOI: http://dx.doi.org/10.3201/eid1904.120903

Predicting Hotspots for Influenza Virus Reassortment

Technical Appendix

Table 1. Human H3N2 influenza virus isolates retrieved from GenBank. We found a total of 632 H3N2 records in China. The virus isolates represented 77 unique geographic locations in 35 prefectures, which we used to model the probability of H3N2 occurrence.

Province	No. H3N2 locations	GenBank LOCUS number(s)
Anhui	3	ACC66805, ADE47294, ADE47534
Beijing	3	AAB06974, ACC67800, ADE47362
Chongqing	2	ACC66832, ADE47551
Fujian	6	ADE46824, ADE48039, ADE47126, ADE47852, ACC66784, AAX63826
Gansu	3	ACC66607, ADE47574, ADE47579
Guangdong	6	AAB06980, ADE48065, ACC77918, ACC66732, AAB66772, ACU82426
Guangxi	3	AAB63703, AF180588, AAB69800
Guizhou	2	AAB69831, ADE46942
Hebei	4	U65672_2, ADE47600, ADE47434, ADE47604
Heilongjiang	2	ADE47446, AAB06975
Henan	2	ADE46750, ADE47973
Hubei	6	ADE47626, ADE47910, ADE48054, ACC67141, ADE48116, AAB06998
Hunan	6	ACC66630, ADE47654, ADE47672 ADE47682, ADE47968, ADE47925
Jiangsu	4	ACC66636, ADE47956, ADE47997, ADD21445
Jiangxi	4	ACC66890, ACC67155, ADE47927, AAB06981
Jilin	1	ACC66470
Liaoning	1	ADE46922
Ningxia	1	AAB63744
Qinghai	1	ADE47778
Shaanxi	1	ACC66962
Shandong	3	AAT64834, ADE47782, AAB69829
Shanghai	2	ACZ05788, ADE47918
Shanxi	1	ADE47832
Sichuan	1	ACC66738
Tianjin	3	ADE46693, ADE47866, ADE47502
Xinjiang	2	ADE47511, ADE47505
Yunnan	1	ADE46812
Zhejiang	3	AAU11522, ADE47908, ACC66703

Table 2. Environmental variables used to predict H3N2 and H5N1 occurrence.

Model	Variable	Source	Reference
H3N2	Human population density	www.ornl.gov/sci/landscan/	(*1*)
	Percent urban	www.fas.harvard.edu/~chgis/data/dcw/	
	Precipitation	www.worldclim.org	(*2*)
	Temperature	www.worldclim.org	(*2*)
H5N1	Chicken density	Marius Gilbert, personal communication	(*3*)
	Duck density	Marius Gilbert, personal communication	(*3*)
	Poultry density*	http://kids.fao.org/glipha/	(*4*)
	Human population density	www.ornl.gov/sci/landscan/	(*1*)
	Percent cropland	http://modis-land.gsfc.nasa.gov/	(*5*)
	Percent water	https://lpdaac.usgs.gov/products/modis_products_table/land_water_mask_derived/land_water_mask_derived/mod44w	(*6*)

*The analysis used poultry density for Egypt because data for chickens and ducks separately was not available.

References

1. Vijayaraj V, Bright EA, Bhaduri BL. High resolution urban feature extraction for population mapping using high performance computing. Proc IGARSS 2007. Barcelona, Spain: IEEE; 2007. p. 278–81.

2. Hijmans RJ, Cameron SE, Parra JL, Jones PG, Jarvis A. Very high resolution interpolated climate surfaces for global land areas. Int J Climatol. 2005;25:1965–78. http://dx.doi.org/10.1002/joc.1276

3. Van Boeckel TP, Prosser D, Franceschini G, Biradar C, Wint W, Robinson T, et al. Modelling the distribution of domestic ducks in Monsoon Asia. Agric Ecosyst Environ. 2011;141:373–80. PubMed http://dx.doi.org/10.1016/j.agee.2011.04.013

4. Franceschini G, Robinson TP, Morteo K, Dentale D, Wint W, Otte J. The Global Livestock Impact Mapping System (GLIMS) as a tool for animal health applications. Vet Ital. 2009;45:491–9. PubMed

5. Hansen MC, Defries RS, Townshend JRG, Sohlberg R. Global land cover classification at 1km spatial resolution using a classification tree approach. Int J Remote Sens. 2000;21:1331–64. http://dx.doi.org/10.1080/014311600210209

6. Carroll ML, Townshend JR, DiMiceli CM, Noojipady P, Sohlberg RA. A new global raster water mask at 250 m resolution. Int J Digital Earth. 2009;2:291–308. http://dx.doi.org/10.1080/17538940902951401

Table 3**.** The number of influenza viruses isolated from swine is much less extensive than the number isolated from humans in China and Egypt. India and Indonesia show a similar pattern (data not shown). Furthermore, the ratios of human to swine isolates in GenBank, FluDB, and the Influenza Virus Resource database are all similar to that of EpiFlu.

	Virus & host	Number of isolates in the EpiFlu database
China	H1N1	
	Human	2336
	Swine	129
	H3N2	
	Human	2950
	Swine	53
	H5N1	
	Human	102
	Swine	9
Egypt	H1N1	
	Human	63
	Swine	0
	H3N2	
	Human	49
	Swine	0
	H5N1	
	Human	171
	Swine	0

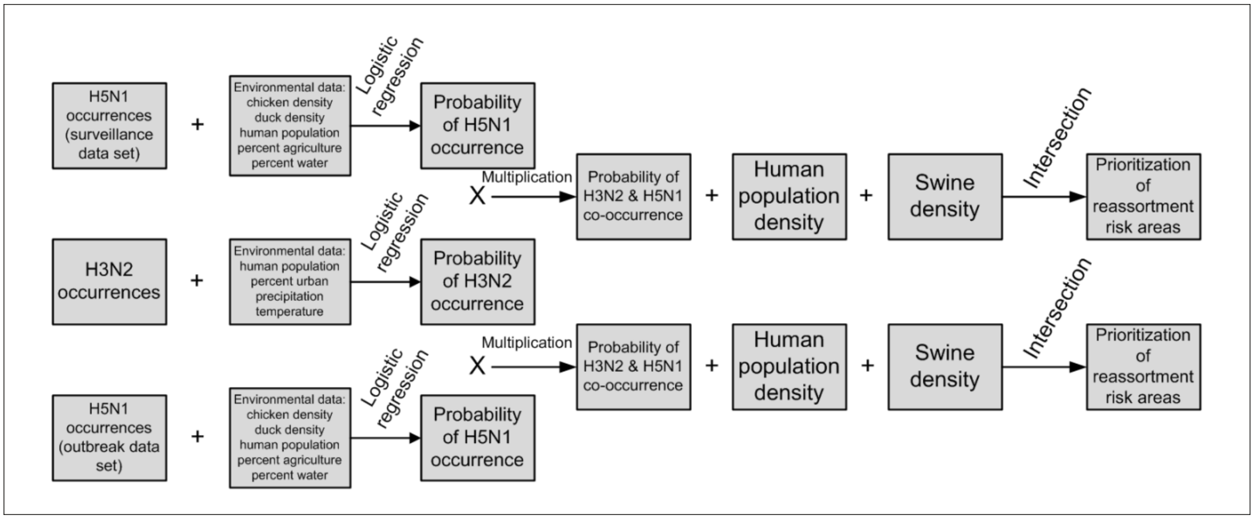

Figure 1. Workflow of the analysis.

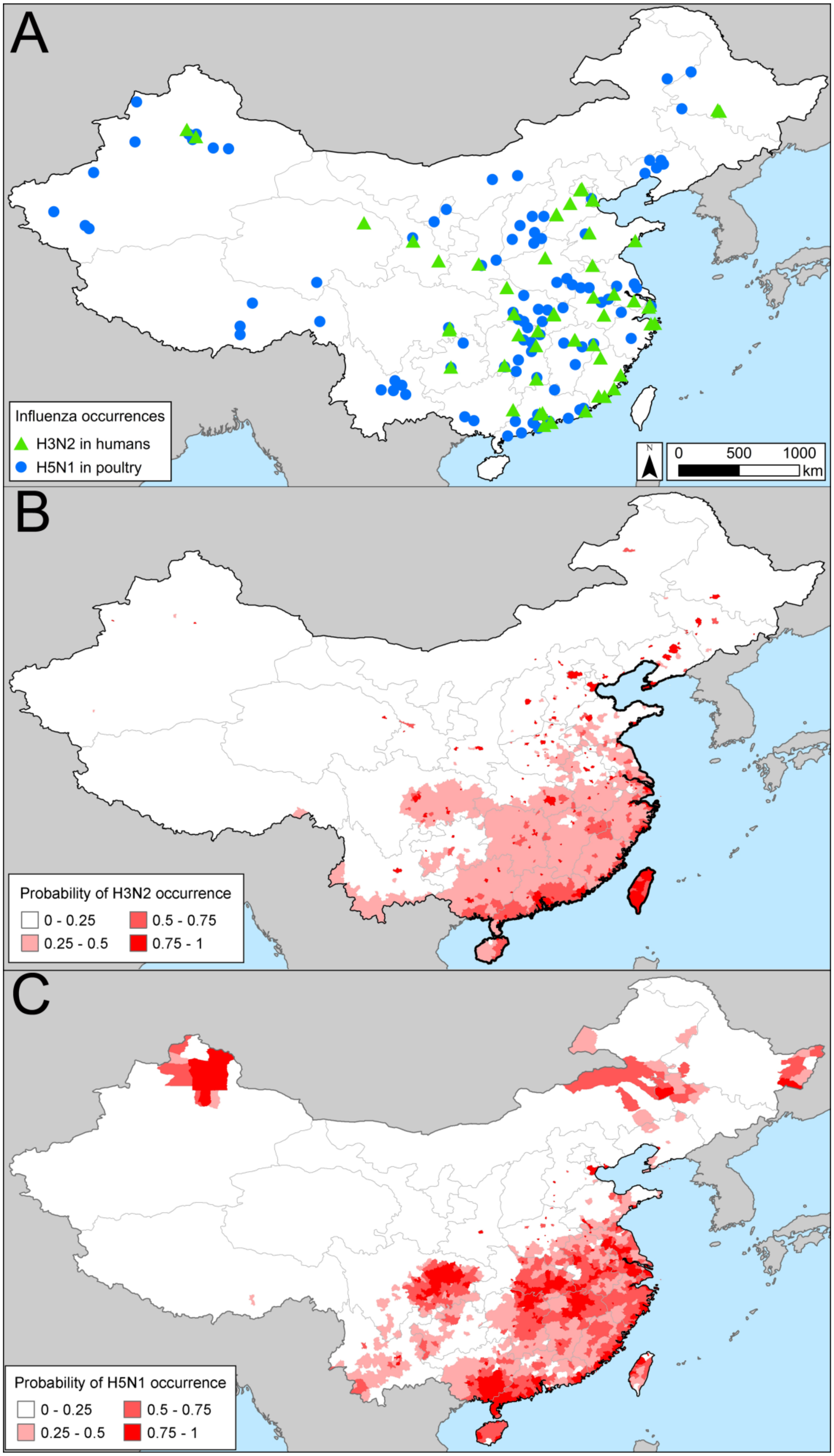
A
Influenza occurrences
H3N2 in humans
H5N1 in poultry
N
0 500 1000
km
B
Probability of H3N2 occurrence
0 - 0.25
0.5 - 0.75
0.25 - 0.5
0.75 - 1
C
Probability of H5N1 occurrence
0 - 0.25
0.5 - 0.75
0.25 - 0.5
0.75 - 1

Figure 2. Influenza risk maps and empirical data using the influenza (H5N1) virus surveillance dataset. A) Cases of subtype H3N2 in humans and H5N1 in People's Republic of China based on active surveillance of wet markets. B) Spatial model of the risk for subtype H3N2 at the prefecture scale generated using logistic regression. C) Map of subtype H5N1 risk constructed using logistic regression with the subtype H5N1 surveillance dataset (see Figure 1 of the article for the risk map constructed from the H5N1 outbreak dataset).

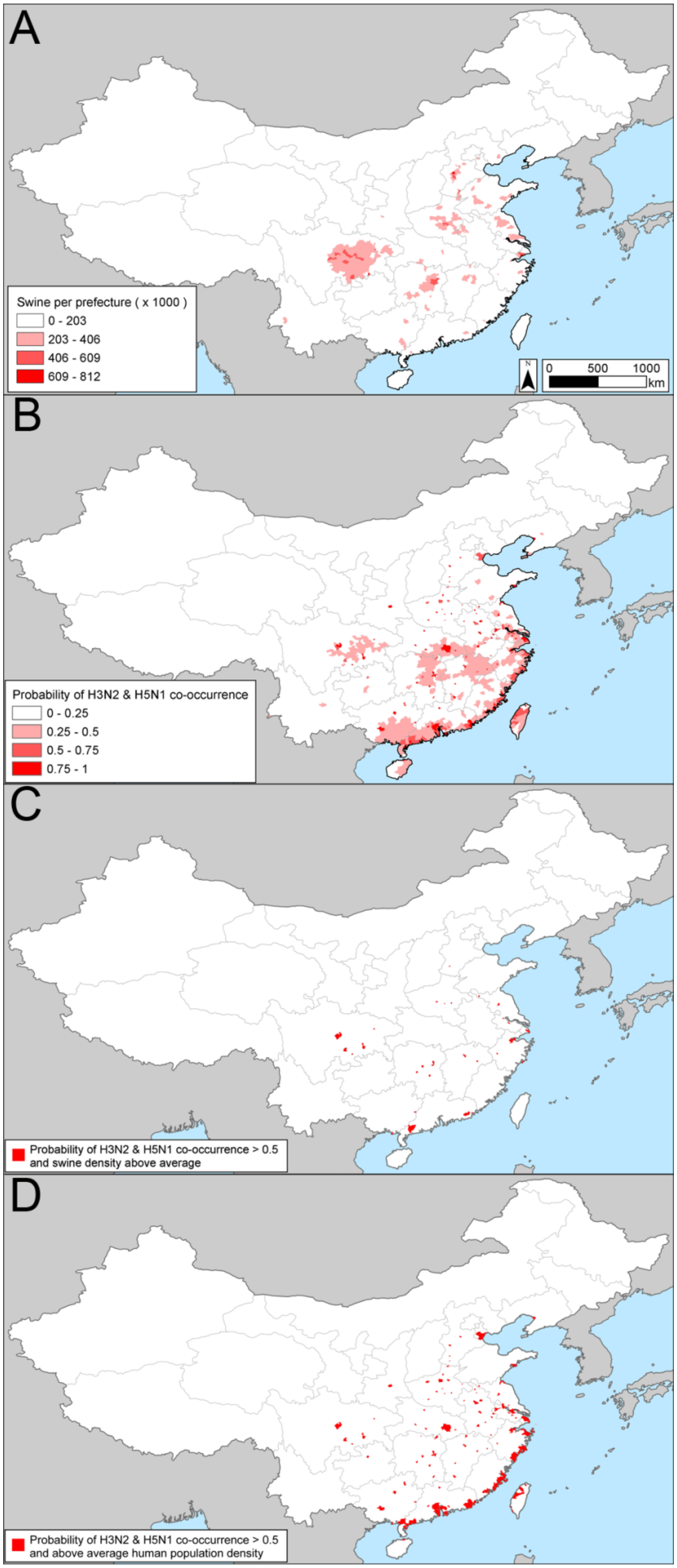
A
Swine per prefecture (x 1000)
0 - 203
203 - 406
406 - 609
609 - 812
N
0 500 1000
km
B
Probability of H3N2 & H5N1 co-occurrence
0 - 0.25
0.25 - 0.5
0.5 - 0.75
0.75 - 1
C
Probability of H3N2 & H5N1 co-occurrence > 0.5
and swine density above average
D
Probability of H3N2 & H5N1 co-occurrence > 0.5
and above average human population density

Figure 3. Prioritization of high-risk areas for influenza reassortment in People's Republic of China based on the influenza virus subtype H5N1 surveillance dataset. A) Density of swine. B) Spatial model of the risk for subtypes H3N2 and H5N1 co-occurrence based on the subtype H5N1 surveillance dataset. C) Areas with a probability of subtype H5N1 and H3N2 co-occurrence >50%, as well as above average swine density. D) Areas with a probability of subtype H5N1 and H3N2 co-occurrence >50%, as well as above average human population density. See Figure 3 of the article for the corresponding models constructed from the H5N1 outbreak dataset.

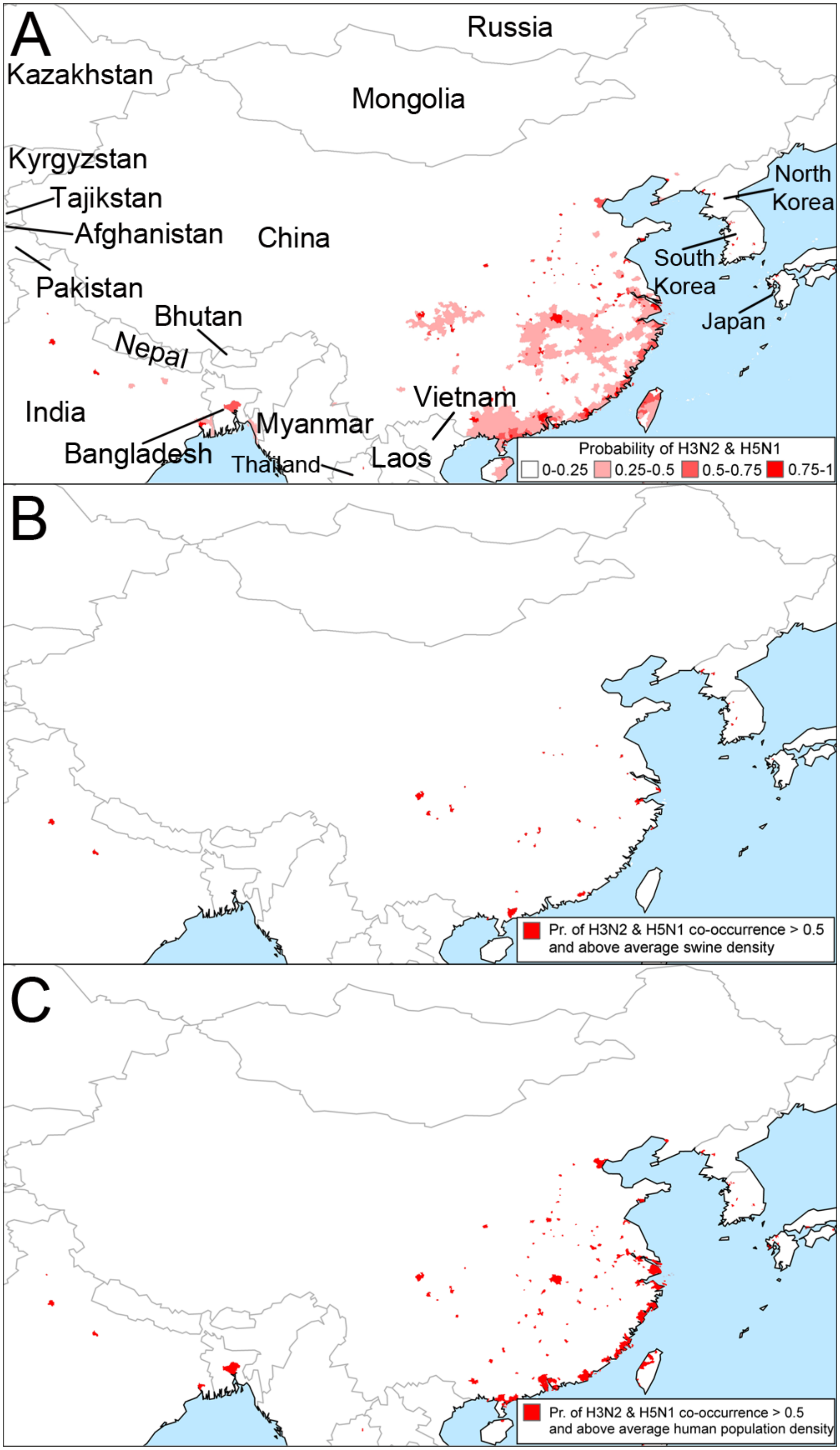
A
Russia
Kazakhstan
Mongolia
Kyrgyzstan
Tajikstan
Afghanistan
China
North Korea
South Korea
Japan
Pakistan
Bhutan
Nepal
India
Bangladesh
Myanmar
Vietnam
Laos
Thailand
Probability of H3N2 & H5N1
0-0.25
0.25-0.5
0.5-0.75
0.75-1
B
Pr. of H3N2 & H5N1 co-occurrence > 0.5
and above average swine density
C
Pr. of H3N2 & H5N1 co-occurrence > 0.5
and above average human population density

Figure 4. Predicted reassortment risk elsewhere in Asia based on the People's Republic of China model using the influenza virus subtype H5N1 surveillance dataset. A) Spatial model of the risk for subtypes H3N2 and H5N1 co-occurrence based on the subtype H5N1 surveillance dataset. B) Areas with a probability of subtype H5N1 and H3N2 co-occurrence >50%, as well as above average swine density. C) Areas with a probability of subtype H5N1 and H3N2 co-occurrence >50% as well as above average human population density. See Figure 4 of the article for corresponding data for the H5N1 outbreak dataset.
